# Supplementary material for: Isthmin-1 (Ism1) modulates renal branching morphogenesis and mesenchyme condensation during early kidney development
Source: Nat Commun. 2023 Apr 25;14:2378. doi: 10.1038/s41467-023-37992-x (PMC10130008; doi:10.1038/s41467-023-37992-x)
Supplement: Supplementary file 1 — Supplementary Information [file 41467_2023_37992_MOESM1_ESM.pdf]

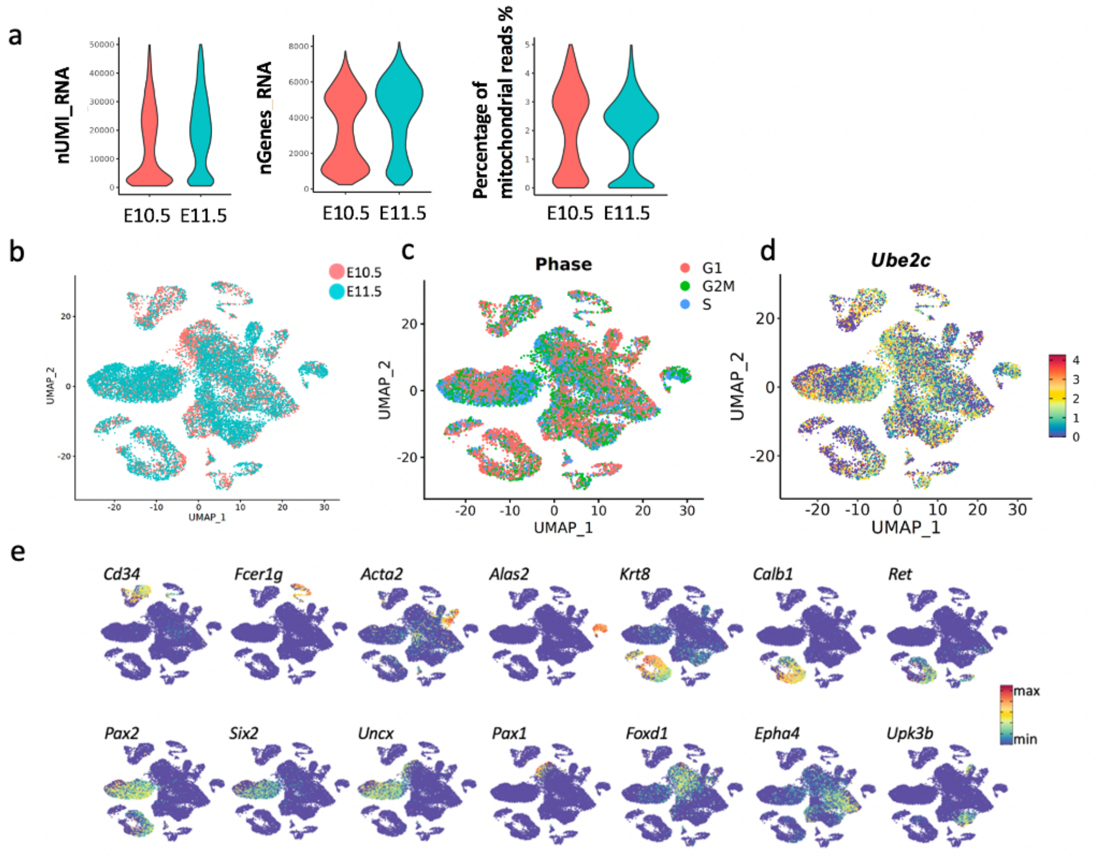

Supplementary Figure 1. Mapping the emergence of kidney branching morphogenesis by sc-RNA sequencing. a, Number of Unique Molecular Identifier (UMI) counts, genes and percentage of mitochondrial reads in E10.5 and E11.5 samples. **b,** Uniform manifold approximation (UMAP) visualization of all the cells sequenced in E10.5 and E11.5 kidney rudiments. **c,** UMAP analysis of merged libraries showed the cells in different cell cycle stages (G1, G2M or S). **d,** Expression of a cell cycle gene *Ube2c* in the UMAP. **e,** UMAP plots depicting expression of different genes in distinct clusters.

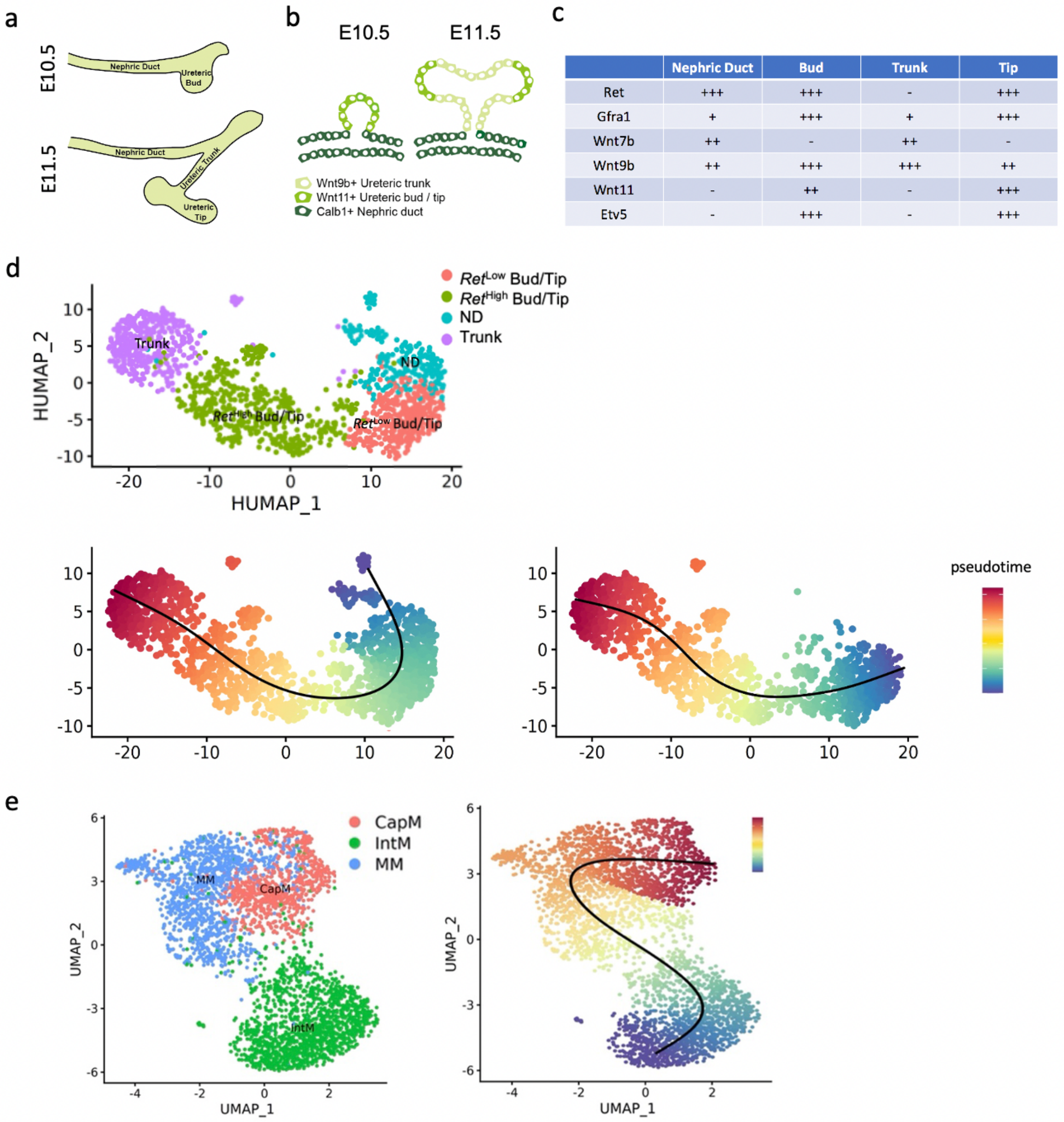

	Nephric Duct	Bud	Trunk	Tip
Ret	+++	+++	-	+++
Gfra1	+	+++	+	+++
Wnt7b	++	-	++	-
Wnt9b	++	+++	+++	++
Wnt11	-	++	-	+++
Etv5	-	+++	-	+++

Supplementary Figure 2. Progression of mouse ureteric epithelial lineage and NPC lineage during early kidney development

a, Schematic diagram showing UE morphology at E10.5 and E11.5. **b,** Schematic diagram showing different ureteric epithelial cell types positive for different markers at E10.5 and E11.5. **c,** A list of accepted markers for ureteric epithelial lineage and their location from ureteric budding to the first branching. **d,** UMAP plots of different cell types in UE lineage from WT embryos. The right panel showed the pseudotime analysis of all UE cells and UE cells without the ND subcluster, respectively, by Sandrine Dudoit's Slingshot. **e,** UMAP plots of different cell types in NPC lineage from WT mice. The right panel showed the pseudotime analysis of NPC lineage by Sandrine Dudoit's Slingshot.

a

Features.plot (E10.5)	Cor	Features.plot (E11.5)	Cor
Gdnf	1	Gdnf	1
Pdgfa	0.989027	Sema3c	0.837722
Timp2	0.987967	Vegfb	0.814288
Sema3f	0.982434	Sema3f	0.798956
Vegfb	0.981306	Fjx1	0.785687
Fjx1	0.980384	Fgf9	0.772838
Sema3c	0.980064	C1qtnf7	0.741731
Ebi3	0.977998	Ism1	0.726302
Ccdc126	0.975935	Ntf3	0.724636
Slit1	0.975594	Gdf11	0.72065
Ism1	0.969696	Grem1	0.71602
Soga1	0.969572	Pcyox1l	0.712262
Gdf11	0.966191	Igfbp2	0.699878
Rspo1	0.963702	Angpt2	0.691389
Fgf10	0.956757	Gpx6	0.689314
Slit2	0.954869	Itih5	0.669875
Pappa	0.953319	Slit1	0.653477
Ntf3	0.949726	Apela	0.64939
Fgf9	0.947867	Kcp	0.626216
Enho	0.946999	Pgf	0.621301

b

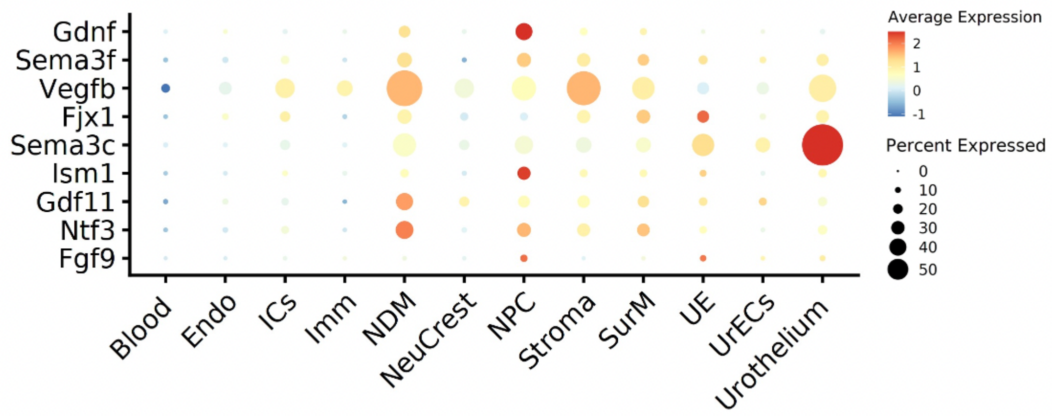

Supplementary Figure 3. Identification of Gdnf-co-expression genes

a, Top 20 gene lists of ECM or ligand proteins in NPC lineage co-expressed with *Gdnf* at E10.5 and E11.5, respectively. See Supplementary Data 1 and 2 for the full list. **b,** Expression pattern of the eight *Gdnf*-coexpression candidates in E10.5 and E11.5 kidney rudiments.

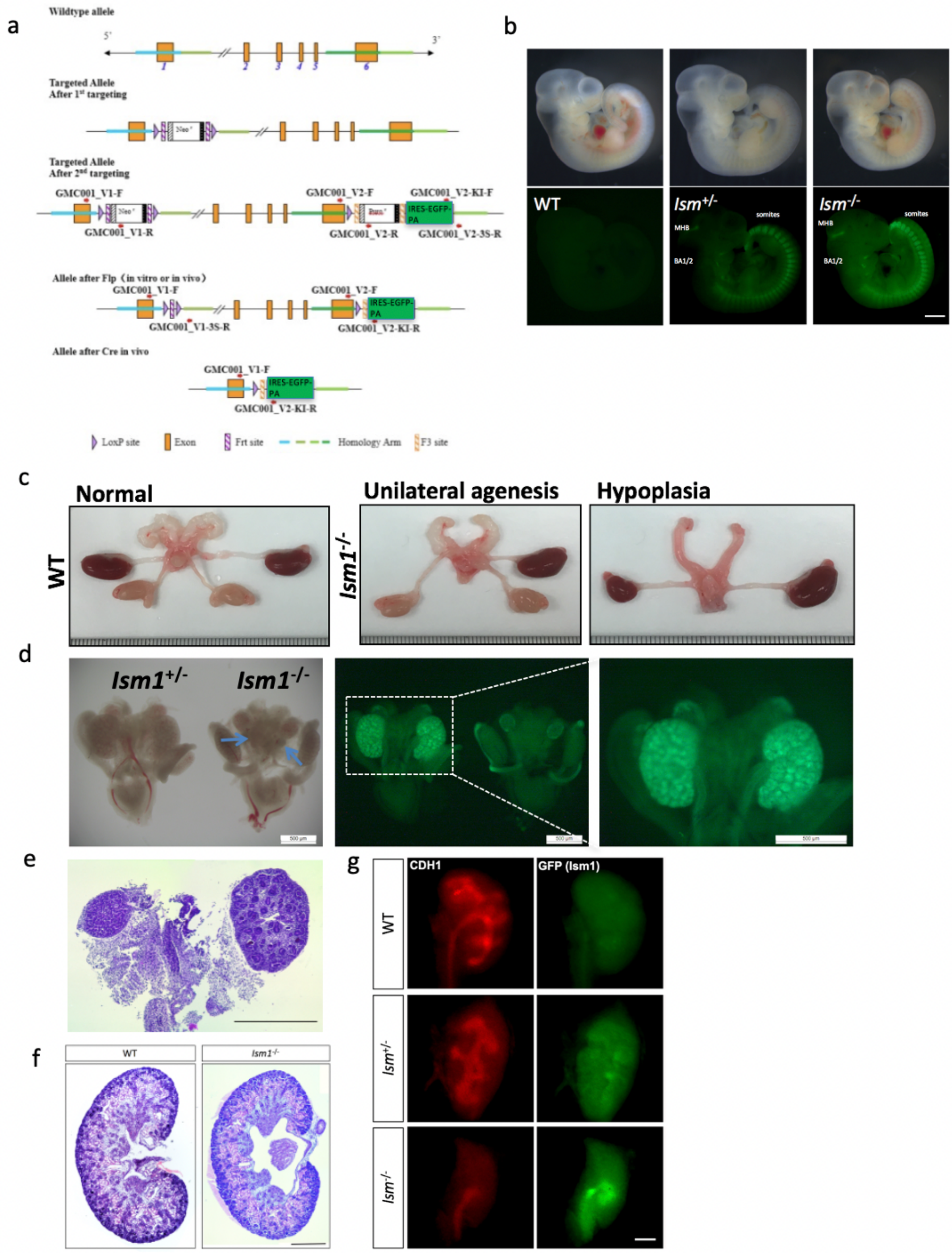

Supplementary Figure 4. Generation of *Ism1*$^{-/-}$ mice and phenotype analysis in kidney. a, Targeting construct of *Ism1* gene for generation of conditional *Ism1* knockout mice and total knockout mice after Cre introduction. **b,** E10.5 embryos of wild-type, *Ism1* heterozygous and homozygous mutants. GFP signal represents *Ism1* expression in heterozygous and homozygous *Ism1* mutant embryos. **c,** Loss of urogenital tract in adult mice with URA. Scale bars, 500 μm. **d-e,** Bilateral (**d**) or unilateral renal agenesis (**e**) in E14.5 *Ism1*$^{-/-}$ embryos. GFP signal represented endogenous *Ism1* expression. Scale bars, 500 μm. **f,** H&E staining of P0 kidneys from

WT and *Ism1*$^{-/-}$ mice. Scale bars, 500 µm. **g,** Whole-mount immunostaining of CDH1 and GFP in kidney rudiments at E12.5, Scale bars, 100 µm. For 4b-g, similar results are observed from at least 3 biologically independent experiments. The penetration of such phenotyps was analyzed in Figure 2b (embryos collected between E14.5 and P0) and Extended Figure13 (E11.5 kidney rudiments).

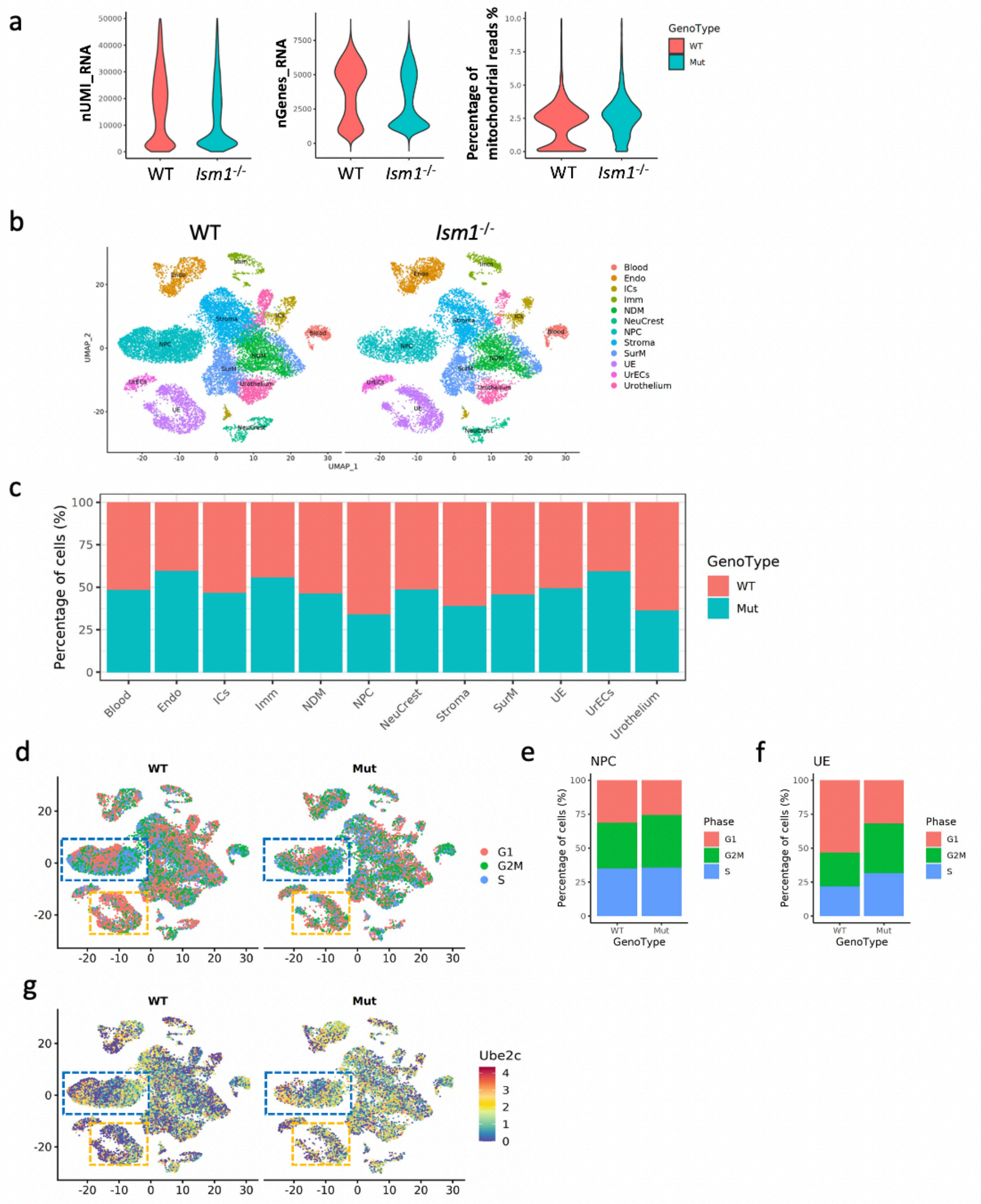

Supplementary Figure 5. Transcriptomic analyses at single-cell resolution in *Ism1*$^{-/-}$ kidney rudiments. a, Number of Unique Molecular Identifier (UMI) counts, genes and percentage of mitochondrial reads in wild-type and *Ism1*$^{-/-}$ kidney rudiments. **b,** UMAP visualization of scRNA-seq in WT and *Ism1*$^{-/-}$ kidney rudiments. **c,** Proportion of cell types in kidney rudiments from both genetypes. **d,** UMAP analysis of merged libraries showing cell cycle stages in WT and *Ism1*$^{-/-}$ kidney rudiments, respectively. **e,** Proportion of NPC cells from different cell cycle stages, in both WT and *Ism1*$^{-/-}$ kidney. **f,** Proportion of UE cells from different cell cycle stages, in both WT and *Ism1*$^{-/-}$ kidney. **g,** Expression of the cell cycle gene *Ube2c* in WT and *Ism1*$^{-/-}$ kidney rudiments, respectively.

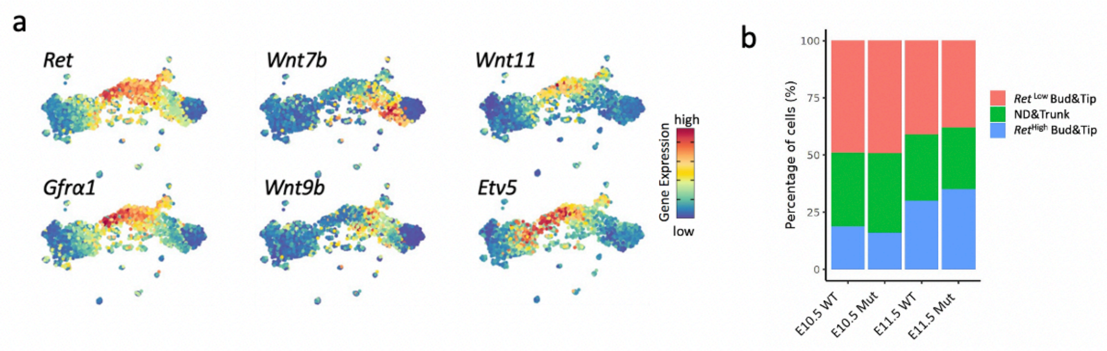

Supplementary Figure 6. Comparison of UE sub-clustering in WT and *Ism1*$^{-/-}$ kidney. a, UMAP plots depicting the expression of different genes. **b,** Proportion of UE sub-clusters from WT and *Ism1*$^{-/-}$ kidney, at both E10.5 and E11.5.

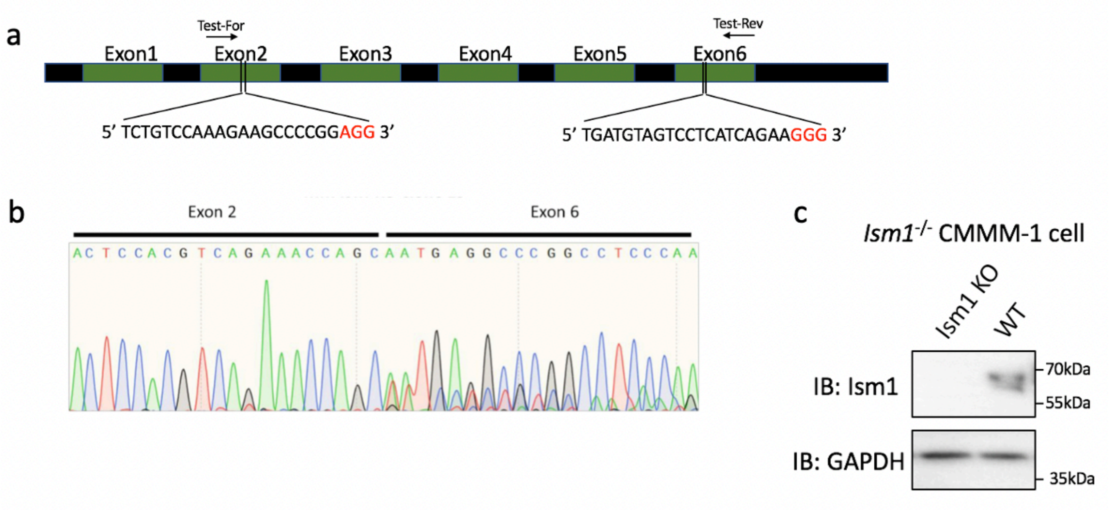

Supplementary Figure 7. Generation of *Ism1*-knockout CMMM-1 cell line. a, Strategy to generate *Ism1*-knockout CMMM-1 cell line by CRSPR/Cas9 gene editing. **b,** Sequencing result to confirm *Ism1* deletion in *Ism1*$^{-/-}$ CMMM-1 cells. **c,** Western blotting of Ism1 expression in WT and *Ism1*$^{-/-}$ CMMM-1 cells. Similar results are observed from at least 3 biologically independent experiments.

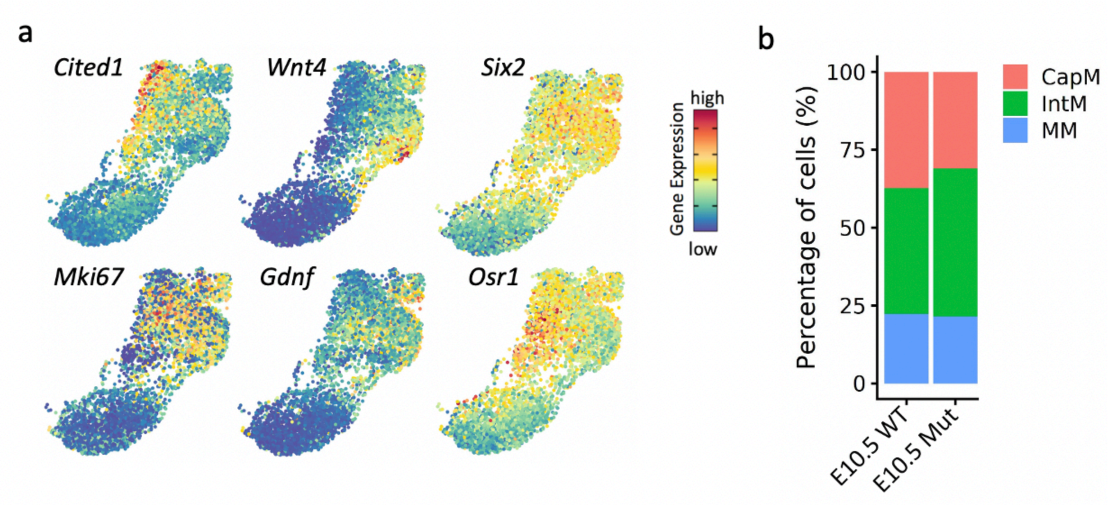

Supplementary Figure 8. Comparison of NPC sub-clustering in WT and *Ism1*$^{-/-}$ kidney. a, UMAP plots depicting the expression of different genes. **b,** Proportion of NPC sub-clusters from WT and *Ism1*$^{-/-}$ kidney, at both E10.5 and E11.5.

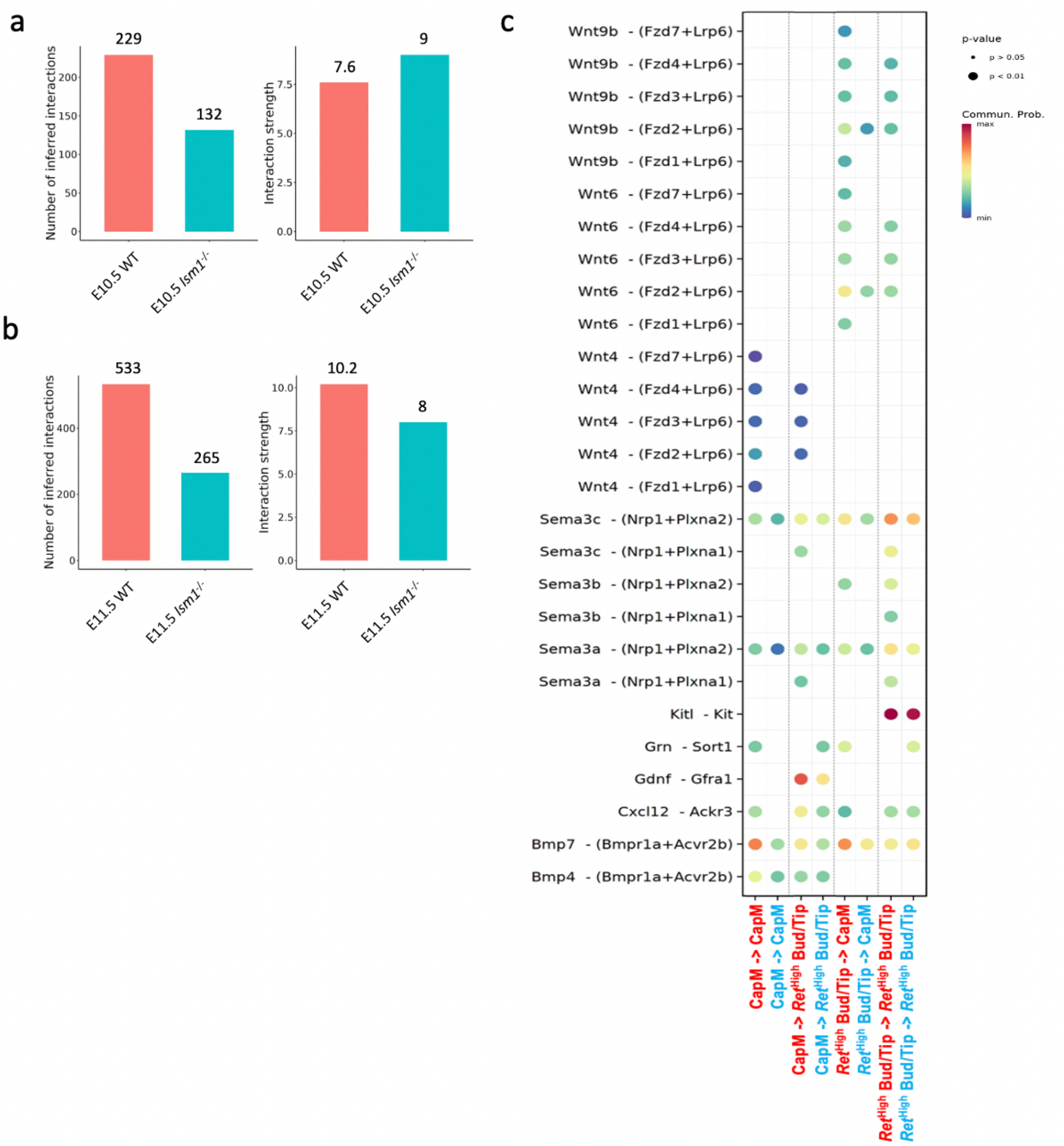

Supplementary Figure 9. Crosstalk analysis in WT and *Ism1*$^{-/-}$ mice and attenuated Gdnf/Ret signaling in *Ism1*$^{-/-}$ mice. a-b, Interaction number and interaction strength between UE and NPC lineage in both WT and *Ism1*$^{-/-}$ kidney rudiments, at E10.5 (a) and E11.5 (b). **c,** Comparison of the crosstalk between UE and NPC lineage in WT and *Ism1*$^{-/-}$ kidney rudiments at E10.5. Dot color represents communication probabilities and dot size represents computed *p*-value. Empty space indicates that the communication probability is zero. *p*-values are computed from one-sided permutation test.

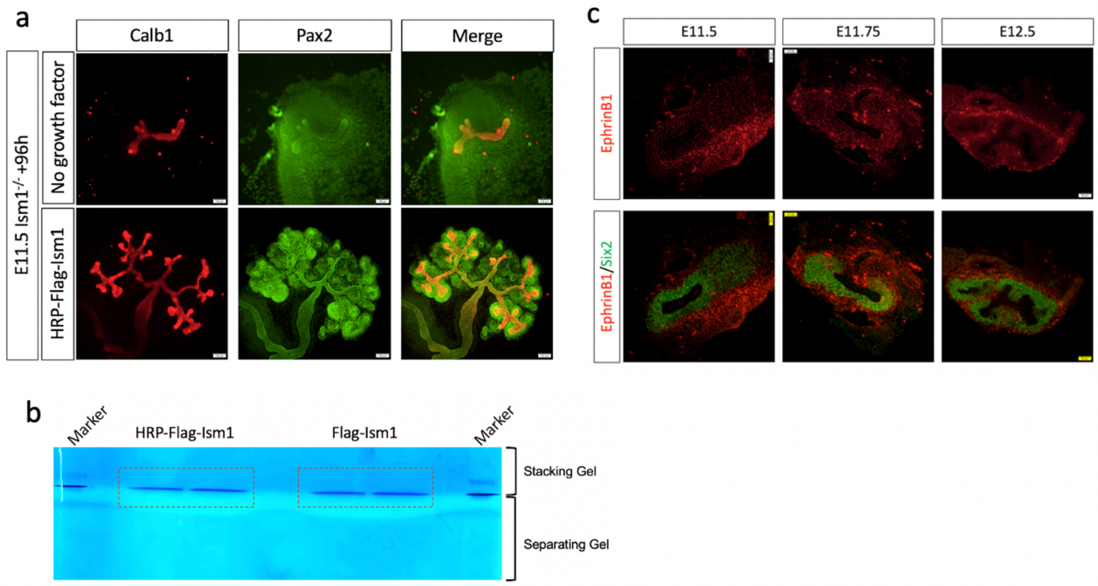

Supplementary Figure 10. HRP-induced proximity labeling in E11.5 kidney tissues. **a,** Validation of the pro-branching function of HRP-conjugated Flag-Ism1 in *Ism1*$^{-/-}$ kidney rudiments. **b,** Coomassie blue staining of proteins pulled down by Neutravidin beads which are potential Ism1-interacted partners. These proteins were run to the boundary of the stacking gel and separating gel and sent for mass spectrometry analysis. **c,** Immunostaining of Ephrin-B1 and Six2 in E11.5 to E12.5 kidney sections from WT mice. Scale bars, 50 μm. Similar results are observed from at least 3 biologically independent experiments.

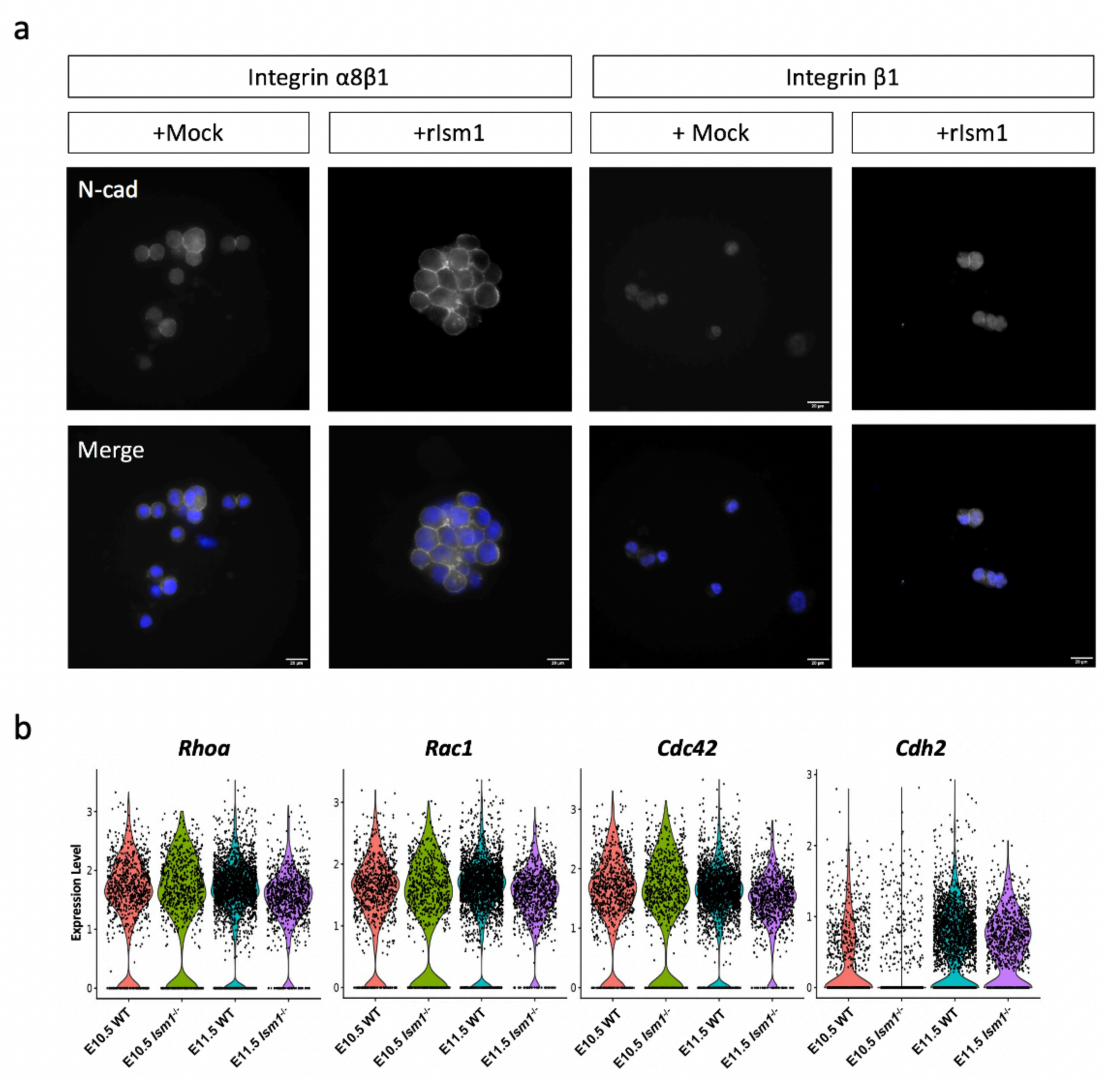

Supplementary Figure 11. Ism1 promotes cell-cell adhesion through Integrin α8β1. a, Immunostaining of N-cadherin in the aggregation assay of HEK293T cells. Cells were transiently transfected with Integrin α8β1 or Integrin β1 followed by rIsm1 treatment. Similar results are observed from at least 3 biologically independent experiments. **b,** Violin plots showing the expression of Integrin-related genes in WT and *Ism1*$^{-/-}$ NPC cells in E10.5 and E11.5 kidney rudiments.

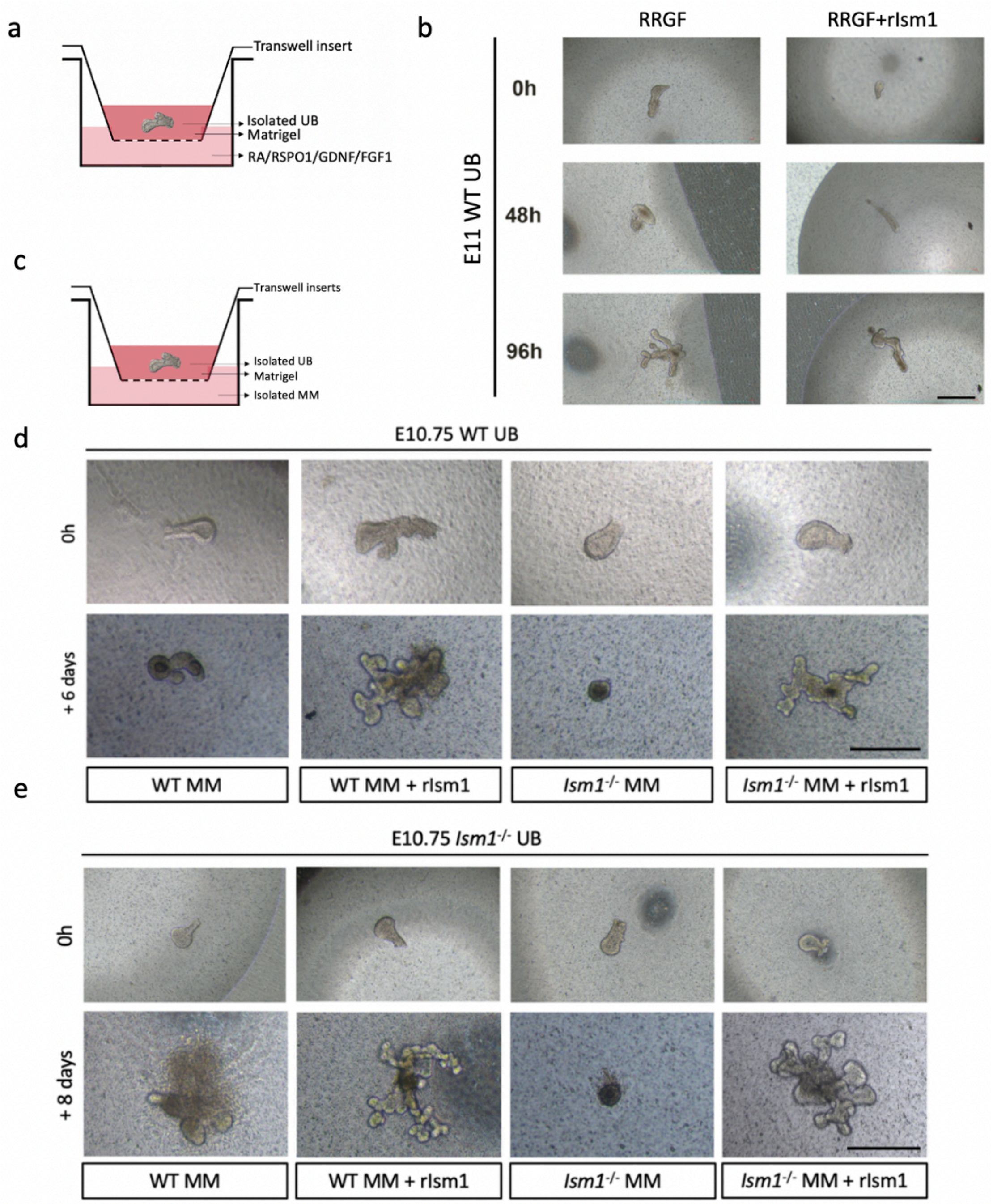

Supplementary Figure 12. Recombination culture using isolated UB and isolated MM in a 3D culture system. **a,** Diagram of the in gel culture of isolated UB. **b,** Isolated E11 WT UB was cultured in gel for 4 days with pro-branching medium (RA, RSPO1, GDNF, FGF1), in the presence or absence of rIsm1. Scale bars, 500 µm. **c,** Diagram of the recombination co-culture assay. Isolated UB was placed in Matrigel in the upper well of a Transwell device and co-cultured, in the presence or absence of rIsm1, with isolated mesenchymal cells plated in the lower wells. **d,** Isolated E10.75 WT UB were co-cultured with either WT or *Ism1*-null MM for 6 days, in the presence or absence of rIsm1. Scale bars, 500 µm. **e,** Isolated E10.75 *Ism1*$^{-/-}$ UB were co-cultured with either

WT or *Ism1*-null MM for 8 days, in the presence or absence of rIsm1. Scale bars, 500 μm. For the in gel culture of isolated UB as shown in (b, d and e), similar results are observed from at least 3 biologically independent experiments.

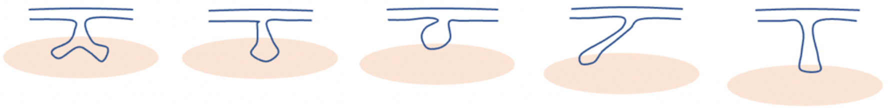

	Type (I)	Type (II)	Type (III)	Type (IV)	Type (V)
WT (n=36)	88.89% (32/36)	11.11% (4/36)	0	0	0
Ism1$^{+/-}$ (n=78)	84.62% (66/78)	15.38% (12/78)	0	0	0
Ism1$^{-/-}$ (n=122)	18.03% (22/122)	19.67% (24/122)	13.11% (16/122)	22.95% (28/122)	26.23% (32/122)

Supplementary Figure 13. Classifications of UB structure phenotypes observed at E11.5. UB structure observed and documented in wild-type, *Ism1*$^{+/-}$ and *Ism1*$^{-/-}$ embryos at E11.5. Class I, UB with T-shaped structure; class II, UB with initiating the first branch with a swelling tip in the mesenchyme; class III, UB initiating the invasion into MM with a structure similar to that at E10.5 stage; class IV, UB reaching to MM peripheral with long and thin UB structure, no obvious tips swelling; class V, UB reaching to MM peripheral with abnormal UB thickening.
