# Supplementary material for: Isthmin-1 (Ism1) modulates renal branching morphogenesis and mesenchyme condensation during early kidney development
Source: Nat Commun. 2023 Apr 25;14:2378. doi: 10.1038/s41467-023-37992-x (PMC10130008; doi:10.1038/s41467-023-37992-x)
Supplement: Supplementary file 3 — Description of Additional Supplementary Files [file 41467_2023_37992_MOESM3_ESM.pdf]

Description of Additional Supplementary Files

File Name: **Supplementary Data 1**
Description: List of Gdnf co-expression ECM or ligand genes in NPC cluster at E10.5.

File Name: **Supplementary Data 2**
Description: List of Gdnf co-expression ECM or ligand genes in NPC cluster at E11.5.

File Name: **Supplementary Data 3**
Description: List of downregulated differentially expressed genes (DEGs) in *Ret*^{High} Bud&Tip sub-cluster in *Ism1*$^{-/-}$ kidney rudiment, compared with that in WT. DEGs between WT and *Ism1*-null kidney rudiments in Supplementary 3/4/5 were calculated with cutoff (Log2FC) >0.25 and *p* value <0.05.

File Name: **Supplementary Data 4**
Description: List of downregulated DEGs in *Ism1*$^{-/-}$ MM sub-cluster compared with that in WT at E10.5

File Name: **Supplementary Data 5**
Description: List of downregulated DEGs in *Ism1*$^{-/-}$ CapM sub-cluster compared with that in WT at E10.5.

File Name: **Supplementary Data 6**
Description: List of primers used in this study.

File Name: **Supplementary Data 7**
Description: Summary of how the mass spectrometry in this study was performed.
