# Supplementary material for: Isthmin-1 (Ism1) modulates renal branching morphogenesis and mesenchyme condensation during early kidney development
Source: Nat Commun. 2023 Apr 25;14:2378. doi: 10.1038/s41467-023-37992-x (PMC10130008; doi:10.1038/s41467-023-37992-x)
Supplement: Supplementary file 5 — Reporting Summary [file 41467_2023_37992_MOESM5_ESM.pdf]

nature portfolio

Corresponding author(s): Zhongjun ZHOU

Last updated by author(s): Mar 6, 2023

Reporting Summary

Nature Portfolio wishes to improve the reproducibility of the work that we publish. This form provides structure for consistency and transparency in reporting. For further information on Nature Portfolio policies, see our Editorial Policies and the Editorial Policy Checklist.

Statistics

For all statistical analyses, confirm that the following items are present in the figure legend, table legend, main text, or Methods section.

n/a	Confirmed	
☐	☒	The exact sample size (*n*) for each experimental group/condition, given as a discrete number and unit of measurement
☐	☒	A statement on whether measurements were taken from distinct samples or whether the same sample was measured repeatedly
☐	☒	The statistical test(s) used AND whether they are one- or two-sided<br>*Only common tests should be described solely by name; describe more complex techniques in the Methods section.*
☒	☐	A description of all covariates tested
☒	☐	A description of any assumptions or corrections, such as tests of normality and adjustment for multiple comparisons
☐	☒	A full description of the statistical parameters including central tendency (e.g. means) or other basic estimates (e.g. regression coefficient) AND variation (e.g. standard deviation) or associated estimates of uncertainty (e.g. confidence intervals)
☐	☒	For null hypothesis testing, the test statistic (e.g. *F*, *t*, *r*) with confidence intervals, effect sizes, degrees of freedom and *P* value noted<br>*Give P values as exact values whenever suitable.*
☒	☐	For Bayesian analysis, information on the choice of priors and Markov chain Monte Carlo settings
☒	☐	For hierarchical and complex designs, identification of the appropriate level for tests and full reporting of outcomes
☐	☒	Estimates of effect sizes (e.g. Cohen's *d*, Pearson's *r*), indicating how they were calculated

Our web collection on statistics for biologists contains articles on many of the points above.

Software and code

Policy information about availability of computer code

Data collection	Illumina HiSeq system to generate sc-RNA sequencing data;<br>Blotting acquisition: Bio-rad software;<br>Confocal image: LSM 800 with Airyscan1;
Data analysis	R statistical software 4.2.2, https://www.r-project.org/<br>Seurat, https://www.r-project.org/<br>CellChat, https://github.com/sqjin/CellChat<br>Pearson correlation coefficient analysis, https://github.com/cran/ccaPP<br>Monocle 2, http://cole-trapnell-lab.github.io/monocle- release/docs/<br>Slingshot, https://github.com/kstreet13/slingshot<br>Image J software (version:2.0.0-rc-43/1.50e)<br>ZEN (blue version) for LSM800 and for LSM980,<br>Graphpad Prism software 9.0

For manuscripts utilizing custom algorithms or software that are central to the research but not yet described in published literature, software must be made available to editors and reviewers. We strongly encourage code deposition in a community repository (e.g. GitHub). See the Nature Portfolio guidelines for submitting code & software for further information.

Data

Policy information about availability of data

All manuscripts must include a data availability statement. This statement should provide the following information, where applicable:

- Accession codes, unique identifiers, or web links for publicly available datasets
- A description of any restrictions on data availability
- For clinical datasets or third party data, please ensure that the statement adheres to our policy

SRA data: PRJNA851535; PRIDE (submission ref No. 1-20230307-71604)

Human research participants

Policy information about studies involving human research participants and Sex and Gender in Research.

Reporting on sex and gender	N/A
Population characteristics	N/A
Recruitment	N/A
Ethics oversight	N/A

Note that full information on the approval of the study protocol must also be provided in the manuscript.

Field-specific reporting

Please select the one below that is the best fit for your research. If you are not sure, read the appropriate sections before making your selection.

☒ Life sciences ☐ Behavioural & social sciences ☐ Ecological, evolutionary & environmental sciences

For a reference copy of the document with all sections, see nature.com/documents/nr-reporting-summary-flat.pdf

Life sciences study design

All studies must disclose on these points even when the disclosure is negative.

Sample size	Sample size was not pre-determined using statistical methods. Experiments were performed with a minimum of 3 independent biological experiments for the analysis and difference. We considered similar research to decide the sample sizes for experiments in this study.
Data exclusions	All criteria for data exclusion in sc-RNA sequencing were established. We removed doublets by R package, with an expected doublet rate of 0.05. In addition, any cells that had either less than 101 UMIs or expression of less than 501 genes, or over 15% UMIs linked to mitochondrial genes are removed.
Replication	Experiments were performed with a minimum of 3 independent biological repeats for the analysis and difference.
Randomization	All the cells from different samples were analyzed as one dataset, and Harmony was applied to integrate different datasets. For experimental samples, they were randomly allocated to control and experimental groups.
Blinding	In this study, the investigators were not blinded when allocating the different genotypes or treatments to avoid contamination between strains or treatments. However, the investigators were blinded for analysis of the data.

Reporting for specific materials, systems and methods

We require information from authors about some types of materials, experimental systems and methods used in many studies. Here, indicate whether each material, system or method listed is relevant to your study. If you are not sure if a list item applies to your research, read the appropriate section before selecting a response.

Materials & experimental systems

n/a	Involved in the study
☐	☒ Antibodies
☐	☒ Eukaryotic cell lines
☒	☐ Palaeontology and archaeology
☐	☒ Animals and other organisms
☒	☐ Clinical data
☒	☐ Dual use research of concern

Methods

n/a	Involved in the study
☒	☐ ChIP-seq
☒	☐ Flow cytometry
☒	☐ MRI-based neuroimaging

Antibodies

Antibodies used

Immunostaining
CALB1 (1:400, C9848, Sigma), SIX2 (1:200, 11562-1-AP, Proteintech), GFP (1:200, ab6556, Abcam), Integrin α8 (1:400, AF4076, R&D), BrdU (1:100, #555627, BD), ETV5 (1:200, 13011-1-AP, Proteintech), phosphorylated-ERK (1:100, #4370, CST), cleaved-Caspase3 (1:200, #9661, CST), anti-DIG-AP antibody (1:2000, 11093274910, Roche), Alexa Fluor 488 donkey anti rabbit IgG (1:500, Invitrogen, A32790), Alexa Fluor 568 donkey anti rabbit IgG (1:500, Invitrogen, A10042), Alexa Fluor 568 donkey anti mouse IgG (1:500, Invitrogen, A10037), Alexa Fluor 488 donkey anti goat IgG (1:500, Invitrogen, A32814), Alexa Fluor 594 donkey anti goat IgG (1:500, Invitrogen, A32758), Alexa Fluor 647 donkey anti rabbit IgG (1:500, Invitrogen, A31573).

Western Blotting
mouse anti-ACTB (1:5000, A5316, Sigma), rabbit anti-GAPDH (1:5000, 10494-1-AP, Proteintech), mouse anti-FLAG (1:2000, F1804, Sigma), rabbit anti-Ism1 (1:5000, Genescript), mouse anti-V5 (1:3000, R960-25, Invitrogen), rabbit anti-phosporylated FAK (1:1000, #3283, CST), rabbit anti-phosphorylated AKT (1:1000, #9271, CST), rabbit anti-phosphorylated ERK (1:1000, #4370, CST), rabbit anti-phosphorylated SRC (1:1000, #2101, CST), mouse anti-N-cadherin (1:2000, #610920, BD), Goat anti-Gdnf (1:2000, AF212, R&D), HRP-conjugated mouse (1:10000, GE Healthcare, NA9310), HRP-conjugated rabbit (1:10000, GE Healthcare, NA9340).

Validation

Beside Ism1, all the other antibodies used in this study were commercial antibodies with validation procedures described on the following manufacturers:
1) CALB1 (1:400, C9848, Sigma)
https://www.sigmaaldrich.com/HK/zh/product/sigma/c9848
Citations (552)
2) SIX2 (1:200, 11562-1-AP, Proteintech)
https://www.ptglab.com/products/SIX2-Antibody-11562-1-AP.htm
Citations (238)
3) GFP (1:200, ab6556, Abcam)
https://www.abcam.com/gfp-antibody-ab6556.html
Citations (1310)
4) Integrin α8 (1:400, AF4076, R&D)
https://www.rndsystems.com/products/mouse-rat-integrin-alpha8-antibody_af4076
Citations (28)
5) BrdU (1:100, #555627, BD)
https://www.bdbiosciences.com/en-us/products/reagents/flow-cytometry-reagents/research-reagents/single-color-antibodies-ruo/purified-mouse-anti-brdu.555627
Citations (281)
6) ETV5 (1:200, 13011-1-AP, Proteintech)
https://www.ptglab.com/products/ETV5-Antibody-13011-1-AP.htm
Citations (16)
7) phosphorylated-ERK (1:100, #4370, CST)
https://www.cellsignal.com/products/primary-antibodies/phospho-p44-42-mapk-erk1-2-thr202-tyr204-d13-14-4e-xp-rabbit-mab/4370
Citations (8434)
8) cleaved-Caspase3 (1:200, #9661, CST)
https://www.cellsignal.com/products/primary-antibodies/cleaved-caspase-3-asp175-antibody/9661?site-search-type=Products&N=4294956287&Ntt=%239661&fromPage=plp&_requestid=1767150
Citations (9997)
9) phosphorylated-FAK (1:200, #3283, CST)
https://www.cellsignal.com/products/primary-antibodies/phospho-fak-tyr397-antibody/3283
Citations (415)
10) anti-ACTB (1:5000, A5316, Sigma)
https://www.sigmaaldrich.com/HK/en/product/sigma/a5316
Citations (4055)
11) rabbit anti-GAPDH (1:50000, 10494-1-AP, Proteintech)
https://www.ptglab.com/products/GAPDH-Antibody-10494-1-AP.htm
Citations (4161)
12) mouse anti-V5 (1:3000, R960-25, Invitrogen)
https://www.thermofisher.com/antibody/product/V5-Tag-Antibody-Monoclonal/R960-25
Citations (1545)
13) rabbit anti-phosphorylated AKT (1:1000, #9271, CST)
https://www.cellsignal.com/products/primary-antibodies/phospho-akt-ser473-antibody/9271?site-search-type=Products&N=4294956287&Ntt=%239271&fromPage=plp&_requestid=1769634

Citations (11029)
14) rabbit anti-phosphorylated SRC (1:1000, #2101, CST)
https://www.cellsignal.com/products/primary-antibodies/phospho-src-family-tyr416-antibody/2101?site-search-type=Products&N=4294956287&Ntt=%232101&fromPage=plp&_requestid=1769726
Citations (879)
15) mouse anti-N-cadherin (1:2000, #610920, BD)
https://www.bdbiosciences.com/en-eu/products/reagents/microscopy-imaging-reagents/immunofluorescence-reagents/purified-mouse-anti-n-cadherin.610920
Citations (504)
16) Goat anti-Gdnf (1:2000, AF212, R&D)
https://www.rndsystems.com/products/human-rat-gdnf-antibody_af-212-na
Citations (60)
17) anti-DIG-AP antibody (1:2000, 11093274910, Roche)
https://www.sigmaaldrich.com/HK/zh/product/roche/11093274910
Citation (1220)
18) HRP-conjugated mouse (1:10000, GE Healthcare, NA9310)
https://www.cytivalifesciences.com/en/us/shop/protein-analysis/blotting-and-detection/blotting-standards-and-reagents/amersham-ecl-hrp-conjugated-antibodies-p-06260
19) HRP-conjugated rabbit (1:10000, GE Healthcare, NA9340)
https://www.cytivalifesciences.com/en/us/shop/protein-analysis/blotting-and-detection/blotting-standards-and-reagents/amersham-ecl-hrp-conjugated-antibodies-p-06260
20) Alexa Fluor 488 donkey anti rabbit IgG (1:500, Invitrogen, A32790)
https://www.thermofisher.com/antibody/product/Donkey-anti-Rabbit-IgG-H-L-Highly-Cross-Adsorbed-Secondary-Antibody-Polyclonal/A32790
21) Alexa Fluor 568 donkey anti rabbit IgG (1:500, Invitrogen, A10042)
https://www.thermofisher.com/antibody/product/Donkey-anti-Rabbit-IgG-H-L-Highly-Cross-Adsorbed-Secondary-Antibody-Polyclonal/A10042
22) Alexa Fluor 568 donkey anti mouse IgG (1:500, Invitrogen, A10037)
https://www.thermofisher.com/antibody/product/Donkey-anti-Mouse-IgG-H-L-Highly-Cross-Adsorbed-Secondary-Antibody-Polyclonal/A10037
23) Alexa Fluor 488 donkey anti goat IgG (1:500, Invitrogen, A32814)
https://www.thermofisher.com/antibody/product/Donkey-anti-Goat-IgG-H-L-Cross-Adsorbed-Secondary-Antibody-Polyclonal/A-11055
24) Alexa Fluor 594 donkey anti goat IgG (1:500, Invitrogen, A32758)
https://www.thermofisher.com/antibody/product/Donkey-anti-Goat-IgG-H-L-Highly-Cross-Adsorbed-Secondary-Antibody-Polyclonal/A32758
25) Alexa Fluor 647 donkey anti rabbit IgG (1:500, Invitrogen, A31573)
https://www.thermofisher.com/antibody/product/Donkey-anti-Rabbit-IgG-H-L-Highly-Cross-Adsorbed-Secondary-Antibody-Polyclonal/A32795

Eukaryotic cell lines

Policy information about cell lines and Sex and Gender in Research

Cell line source(s)	Mouse metanephric mesenchyme (CMMM-1, #W501) Probetex<br>Mouse ureteric bud (CMUB-1, #W508) Probetex<br>HEK293T cells (ATCC)
Authentication	Mouse metanephric mesenchyme (CMMM-1, #W501) and mouse ureteric bud (CMUB-1, #W508) obtained from Probetex, were maintained in DEME (high glucose) medium with 10% FBS. HEK293T cells were maintained in DEME (high glucose) medium with 10% FBS. No specific techniques/procedures were used for the authentication of the cell lines.
Mycoplasma contamination	All cell lines used were tested negative for mycoplasma.
Commonly misidentified lines (See ICLAC register)	No commonly misidentified cell lines were used.

Animals and other research organisms

Policy information about studies involving animals; ARRIVE guidelines recommended for reporting animal research, and Sex and Gender in Research

Laboratory animals	All mice were on a C57BL/6J background raised at 22 °C, 50-60% humidity in the normal 12/12 light-dark cycle, and were fed a conventional diet. Actb-Cre males (8-12 week-old) were crossed with 8-week-old Ism1-floxed female mice to generate heterozygous and homozygous Ism1-CKO;Actb-Cre mice. Both homozygous and heterozygous Ism1-CKO;Actb-Cre mice were normal in reproduction system, thus they were used for obtaining Ism1+/- or Ism-/- embryo. The noon of the day on which a vaginal plug was found was designated as E0.5. To obtain embryo samples from the different stages, the pregnant females were sacrificed by cervical dislocation, and the embryos were dissected in PBS buffer on ice.
Wild animals	No wild animals were used in the study.

Reporting on sex	Male and female were both included in random numbers per experiment.
Field-collected samples	The study did not involve samples collected from the field.
Ethics oversight	Ethical oversight was provided by the University of Hong Kong, Hong Kong SAR, China

Note that full information on the approval of the study protocol must also be provided in the manuscript.
